# Normative Topographic Anterior and Posterior Corneal Astigmatism: Axis Distribution and Its Relations with Ocular and Biometric Parameters

**DOI:** 10.3390/jcm12113664

**Published:** 2023-05-25

**Authors:** Ignacio Almorín-Fernández-Vigo, Inés Sánchez-Guillén, José Ignacio Fernández-Vigo, Bárbara Burgos-Blasco, Lucía De-Pablo-Gómez-de-Liaño, José Ángel Fernández-Vigo, Ana Macarro-Merino

**Affiliations:** 1 Centro Internacional de Oftalmología Avanzada, 06010 Badajoz, Spain; 2 Department of Ophthalmology, Hospital Perpetuo Socorro, 06010 Badajoz, Spain; 3 Centro Internacional de Oftalmología Avanzada, 28010 Madrid, Spain; 4 Department of Ophthalmology, Hospital Clínico San Carlos, 28040 Madrid, Spain; 5 Department of Ophthalmology, Hospital 12 de Octubre, 28041 Madrid, Spain; 6 School of Medicine, Universidad de Extremadura, 06006 Badajoz, Spain

**Keywords:** corneal astigmatism, posterior astigmatism, refractive surgery, axis distribution

## Abstract

Corneal astigmatism correction is a key factor when planning refractive treatment for ametropies with intraocular lenses. We aim to obtain normative anterior and posterior corneal astigmatism (ACA and PCA, respectively) data in a local population and to describe their axis distribution and their association with other parameters. A total of 795 patients with no ocular diseases were evaluated with corneal tomography and optical biometry. Only data of the right eye were included. Mean ACA and PCA were 1.01 ± 0.79 and 0.34 ± 0.17 D, respectively. Vertical steep axis distribution was 73.5% for ACA and 93.3% for PCA. Axis orientation between ACA and PCA matched best for vertical orientation (especially 90° to 120°). Vertical ACA orientation frequency decreased with age, with a more positive sphere and less ACA. Vertical PCA orientation frequency increased with higher PCA. Eyes with vertical ACA orientation were younger and showed a greater white-to-white (WTW) measurement, anterior corneal elevations, ACA and PCA. Eyes with vertical PCA orientation were younger and showed greater anterior corneal elevations and PCA. Normative ACA and PCA data in a Spanish population were presented. Steep axis orientations presented differences with age, WTW, anterior corneal elevations and astigmatism.

## 1. Introduction

The increase in corneal refractive surgeries by laser subtraction and/or implantation of more sophisticated intraocular lenses requires detailed knowledge of the ocular structures, particularly the cornea and its optical properties. Corneal astigmatism appears when the dioptric power of the cornea is not equally distributed along its meridians, with flatter and steeper meridians usually oriented in an orthogonal manner.

**Figure 1:**
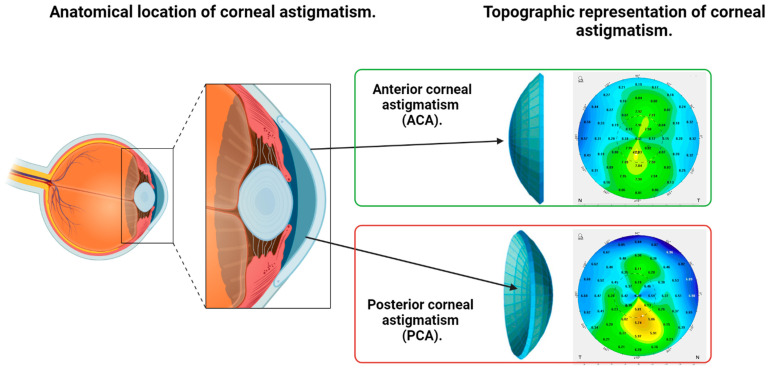
Illustration of the anatomical location of anterior (ACA) and posterior corneal astigmatism (PCA) and their topographic representation as shown by a Scheimpflug tomography.

Corneal astigmatism correction is a key factor when planning refractive treatment for ametropies with intraocular lenses [1,2,3,4,5]. Anterior corneal astigmatism (ACA) is the main source of corneal astigmatism (Figure 1), but astigmatism arising from the posterior surface of the cornea (PCA), although usually small in magnitude, should also be taken into account to better estimate the real (total) corneal astigmatism [6,7,8,9,10,11,12,13].

To measure the refractive power and the characteristics of the cornea, its shape (curvature, measured in millimetres (mm)) must be first determined. Then, using a refractive index, those measurements can be converted into power, which is measured in dioptres (D).

Several systems based on different principles have been used for that purpose. The former Javal-type keratometer uses the reflection method: it measures the size of a reflected image by the cornea from an illumination source. To do those calculations, some optical properties which do not reflect the reality of all the corneas must be assumed. However, the main limitation of this method is that it only provides the central power of the anterior cornea. In addition, its measurements depend on tear film stability [14]. Nevertheless, due to its simplicity and good repeatability [15,16], this device is still used in most clinical settings.

Later, the reflection topography expanded the measurement area by using multiple ring-shaped illumination sources (known as Placido discs) which became the most popular device, and also because of the computerization of the data. These devices allowed topography maps of a greater area of the anterior cornea to be obtained. The possibility of evaluating corneal power with high repeatability in different areas opened the opportunity to analyse corneal topography patterns [14], but still only the anterior cornea could be measured.

The introduction of new technology based on obtaining b-scans of the cornea (tomography) allowed clinicians to also evaluate the posterior cornea in their daily practice and not only in laboratory or investigative settings. The first commercially available device combined horizontal scanning slit technology with a Placido disc topographer and a fixed central camera. Several tomographic images of the cornea are acquired after projecting a slit beam of light from one side of the cornea to the opposite in a scanning horizontal pattern. Measuring both anterior and posterior corneal power in a wide area was then possible. Many clinical studies evaluating clinical applications of this new technology were reported; some of them detected that posterior cornea accuracy was not always perfectly reliable, especially in post-operative corneas [17].

The next commercially available device was also a tomographer. The innovation lied in a centrally located slit that rotates simultaneously with a peripheral camera. The slit rotates to produce an asterisk or radial pattern of scans, which are captured by the peripheral camera. The camera is incorporated following the Scheimpflug principle to avoid the defocus effect from images taken from a tilted camera. The main advantages of this method are that all of the single scans are nearly centred at the slit centre of rotation, so diminishing movement artefacts is easier, and that the vast majority of data comes from the central cornea, which is the most important area [14].

Rotating Scheimpflug tomography has remained the gold standard for posterior corneal power measurement and evaluation for several years because of its good accuracy [16,18,19,20] even in irregular corneas, such as post-operative [20] or ectatic corneas [21]. The main limitation still remains in media opacity, which significantly limits penetration of the emitted slit blue light which could lead to artefacted power measurements.

Recently, other devices have emerged to address measuring the posterior cornea. A device based on former reflection topography with a novel specific “light-emitting-diode”-based reflection spatial organization was able to evaluate posterior corneal power but in a limited ring [13]. Additionally, optical coherence tomography technology has been recently applied to the measurement of posterior corneal power, with some advantages over Scheimpflug imaging due to its deeper penetrance in media opacity but still with no superiority over the previous technology in terms of repeatability [20].

The first studies that experimentally quantified posterior astigmatism used Purkinje images obtained with Polaroid cameras, as in Royston and Dunne’s work [6]. With the appearance of the aforementioned tomographs, several population studies emerged. In a healthy population, Dubbelman reported a PCA of 0.33 ± 0.01 D with a manual Scheimpflug camera [22]. Since then, several studies have described ACA and PCA in large populations from different locations [9,10,12,13,23,24,25,26,27]. Furthermore, the PCA steep axis orientation was assessed and correlated with ACA steep axis orientation in several works, even detecting trends with some parameters, including age. These normative data helped to confirm, and even improve, some of the previous algorithms that empirically considered a fixed amount of PCA when correcting corneal astigmatism with toric intraocular lenses to diminish the residual refractive error instead of measuring PCA. Due to the possibility of measuring PCA, one of the main previous sources of a calculation error, the popularity of toric intraocular lenses has also increased in recent years.

However, the study of the ACA and PCA axis distribution and its relationship with biometric variables is not extensive. This study was designed to obtain normative ACA and PCA values in a Spanish population, their axis distribution and their association with other parameters.

## 2. Materials and Methods

In this cross-sectional study, participants were consecutively recruited among those visiting the Centro Internacional de Oftalmología Avanzada (CIOA) in Madrid, Spain, for a routine ophthalmologic exam over the period 1 November 2012, to 30 June 2013. The study protocol adhered to the tenets of the Declaration of Helsinki and received Review Board approval from the CIOA. Written informed consent was obtained from all participants.

**Figure 2:**
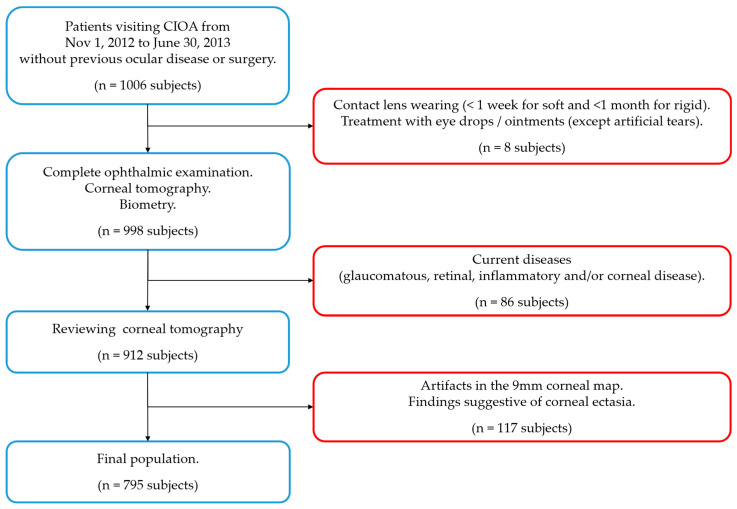
Flow chart for inclusion (blue squares) and exclusion criteria (red squares). *n* = number of subjects.

The inclusion criteria were no history of ocular disease or surgery. The exclusion criteria were one or more of the following situations (Figure 2): contact lenses wearing close to the examination (less than 1 week for soft contact lenses and less than 1 month for rigid contact lenses), topical treatment (except for artificial tears) as wells as a glaucomatous, retinal, inflammatory and/or corneal disease (congenital or acquired) with a previous diagnosis or diagnosed during the examination. Tomographic exclusion criteria indicating a possible ectasia were also used: (a) asymmetric bow-tie pattern or abnormal steepening or skewed radial axis [28] or (b) the combination of a rate of progression of pachymetry (RPI) of more than 1.2 with a thinnest point less than 450 µm combined with a posterior elevation greater than 13.5 µm [29].

A complete ophthalmic examination was performed on all subjects, including visual acuity, manifest refraction, tonometry, anterior segment biomicroscopy, corneal tomography (Pentacam, Oculus Optikgeräte GmbH), biometry (IOLMaster 500, Carl Zeiss, Meditec) and a fundus exam following pupil dilation. Only data of the right eye of each subject were included in this study.

Corneal tomography was performed with the Pentacam single rotation Scheimpflug camera with a capture rate of 25 B-scans in 2 s. Only those exams with sufficient quality and at least 9 mm without extrapolations were included.

Variables included in the study were age, sex and subjective refraction in diopters (D) (sphere and refractive astigmatism (RA) with its steep axis) from the medical record. Pupil diameter (PD) was measured in millimetres (mm); anterior chamber depth (ACD) was measured from the endothelium in mm; anterior and posterior corneal elevations at the apex and thinnest locations (Ele F Apex, Ele B Apex, Ele F Thin, Ele B Thin) were measured in microns (µm); thinnest pachymetry (Pachy) was measured in mm; ACA and PCA were measured in a 3 mm diameter ring centred on the apex in D and their steep (major) axis; anterior and posterior flat (K1F and K1B), steep (K2F and K2B) and mean keratometry (KmF and KmB) were measured in a 3 mm diameter ring centred on the apex in D; and anterior and posterior corneal asphericity were measured in a 6 mm diameter zone (QF and QB) from the Pentacam. Axial length (AXL) and WTW distances in mm were obtained from the IOLMaster 500.

The steep axis of corneal astigmatism was extracted from the Pentacam. Astigmatisms at 180° were recorded as 0°, with the definitive range being 0 to 179.9°. The categorization of the astigmatisms was as follows: horizontal (0° to 30° and 150° to 179.9°), vertical (60° to 120°) and oblique (30.1° to 59.9° and 120.1° to 149.9°).

*Statistical analysis.* The number and percentage of cases in each category (qualitative variables) and mean ± standard deviation (SD), range and percentiles (quantitative variables) were used for descriptive analysis. The Kolmogorov–Smirnov test was used to evaluate the normal distribution of the data. Differences in parameters between groups were investigated using the Kruskal–Wallis test. The analysis of variance (ANOVA) was used to determine if there was a statistically significant difference between categorical groups by testing for differences of means using variance. Significance was set at *p* ≤ 0.05. The data were analyzed using the Statistical Package for the Social Sciences for Windows (SPSS v. 25, IBM Corp. Armonk, NY, USA).

## 3. Results

The final study population consisted of 795 right eyes from 795 patients. Of the patients, 63.6% were female and 50.8% were hyperopic. All participants were Caucasic.

The mean, SD and range of the variables are presented in Table 1. Supplemental Figure S1 shows the frequency distribution of RA, ACA and PCA.

Steep axis orientation (vertical, oblique and horizontal) distribution was 254 (39.4%), 94 (14.6%) and 296 (46%) for RA; 584 (73.5%), 113 (14.2%) and 98 (12.3%) for ACA; and 742 (93.3%), 42 (5.3%) and 11 (1.4%) for PCA, respectively.

**Figure 3:**
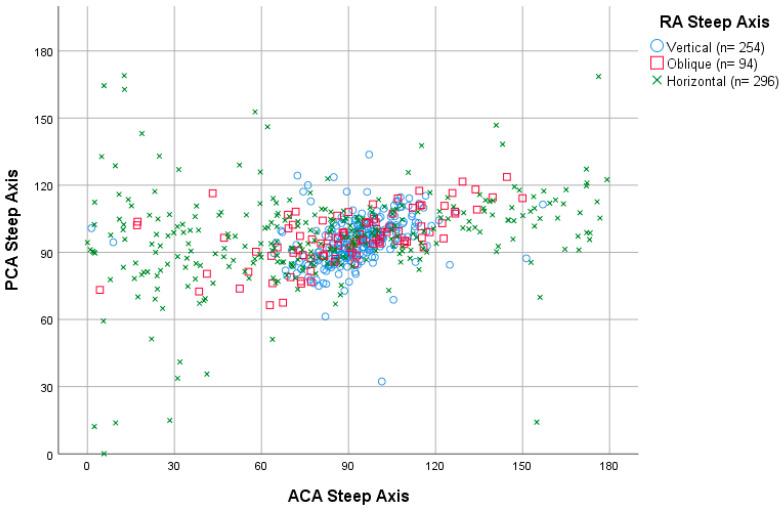
Dispersion diagram of ACA (*n* = 795) and PCA (*n* = 795) steep axis categorized by RA (*n* = 644) steep axis. ACA = anterior corneal astigmatism; PCA = posterior corneal astigmatism; RA = refractive astigmatism; *n* = number of subjects.

The relation between the axis orientation of ACA and PCA is shown in Figure 3. The best coincidence on axis orientation between ACA and PCA was for the vertical steep axis orientation (60° to 120°), where nearly all the cases were included. Within this orientation, the range of 90° to 120° had the least dispersion. Additionally, a trend was seen for the oblique ACA axis, in which most of the ACA axis range of 120° to 180° (nasal zone) corresponded to a PCA axis range of 90° to 120°. The horizontal ACA axis range of 0° to 30° had the highest dispersion.

**Figure 4:**
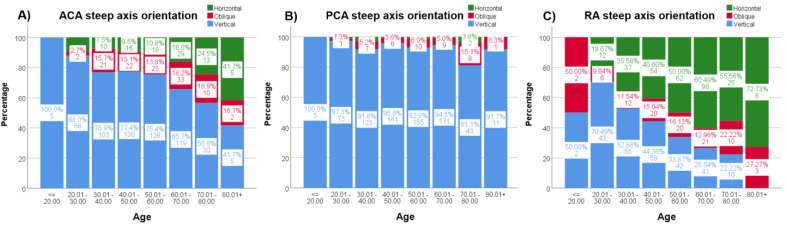
(**A**–**C**). Percentages of the steep axis distribution through decades for ACA (**A**), PCA (**B**) and RA (**C**). Each column shows the percentage (%) and the number of subjects for the three steep axis orientations (green = horizontal, red = oblique and blue = vertical) and for every column. ACA = anterior corneal astigmatism; PCA = posterior corneal astigmatism; RA = Refractive astigmatism.

**Figure 5:**
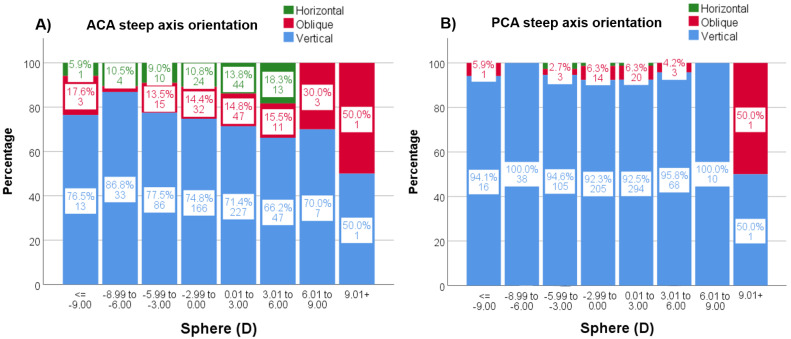
(**A**,**B**). Percentages (%) and n values of the steep axis distribution by sphere for ACA (**A**) and PCA (**B**). ACA = anterior corneal astigmatism; PCA = posterior corneal astigmatism.

ACA steep axis distribution showed a statistically significant tendency (*p* < 0.001) to decrease the frequency of vertical steep axis orientation while increasing the horizontal orientation throughout age decades (Figure 4A). This tendency did not reach statistical significance (*p* = 0.051) for the PCA axis distribution (Figure 4B). RA also shifted from vertical (with the rule, WTR) to horizontal (against the rule, ATR) steep axis orientation with decades (*p* < 0.001) (Figure 4C). The same, but weak, tendency for vertical axis orientation to be less frequent with increasing spheres was seen for the ACA (*p* = 0.040), especially from −9 D, but not for the PCA (*p* = 0.355) (Figure 5).

**Figure 6:**
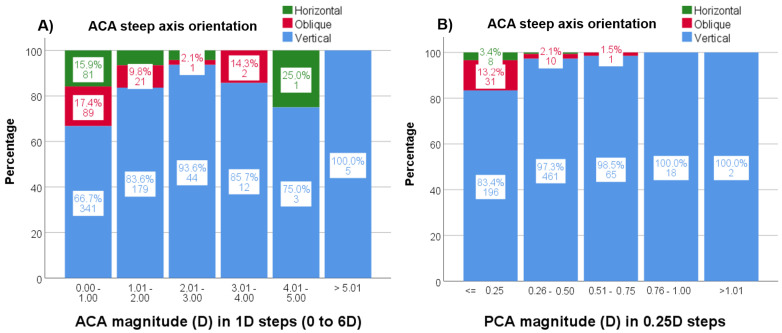
(**A**,**B**). Percentages (%) and n values of the steep axis distribution by astigmatism magnitude for ACA (**A**) and PCA (**B**). ACA = anterior corneal astigmatism; PCA = posterior corneal astigmatism.

In addition, with increasing ACA magnitude, the frequency of vertical ACA steep axis orientation increased up to 3 D astigmatism (*p* < 0.001) but not for higher magnitudes (Figure 6A). Lastly, with increasing PCA magnitude, the frequency of PCA vertical steep axis orientation increased (*p* < 0.001) (Figure 6B).

Supplemental Tables S1 and S2 show the results of the ANOVA test for the differences between the three steep axis astigmatism orientations (vertical, oblique and horizontal) about the different variables compared for ACA and PCA, respectively.

## 4. Discussion

Given the advances in corneal and cataract surgery, the study of corneal characteristics and the effect of biometric variables have increasing relevance. A normative database in a Spanish population with a thorough analysis of ACA and PCA is hereby presented, detailing axis distribution and its association with other parameters.

Normative PCA and/or ACA measurements from the population studies found in the literature are presented in Table 2. In one of the largest population studies to date, Hoffman described a mean ACA of 0.98 ± 0.78 D using the IOL Master in 23,239 eyes of 15,448 Caucasic individuals [23]. In our study, we obtained a mean ACA of 1.01 ± 0.79 D, with a range of 0 to 5.80 D, similar to other population studies in the literature [9,12,25,30]. Mean ACA measurements from those studies range from 0.80 [27] to 1.35 D [26]. Furthermore, we estimated PCA at 0.34 ± 0.17 D (0 to 1.10), which was very similar to those population studies. Regardless of the device and the image capture methodology used, the PCA measurements exhibited lower variability between studies with different racial populations, ranging from 0.30 [9,10] to 0.37 [13].

Astigmatism steep axis orientation has been widely described in the literature, being predominantly vertical for RA, ACA and PCA (Supplemental Table S3). [7,9,11,12,23,25] This corresponds to a WTR for RA and ACA but to an ATR for PCA because of the convergent and divergent optical configurations for the anterior and posterior cornea, respectively [31]. For horizontal and oblique steep axis orientations, some studies showed a similar distribution, while others found the horizontal orientation to be more frequent for ACA or PCA [11,23,25,32]. This might be explained because of the differences in population characteristics such as age, race, sphere and ACA magnitude.

In the present study, a tendency of increased vertical steep axis orientation with an ACA magnitude up to 3 D and a tendency to decrease this axis orientation with a more positive sphere (from −9 D onwards) were noted, but only for ACA and not for PCA. In addition, a progressive decrease in the vertical steep axis orientation has been described for ACA [10,26] throughout the decades, particularly from the sixth, while increasing the horizontal and oblique orientations [7,9,12,22,23,33,34]. In the present study, ACA oblique orientation remained more frequent than horizontal from the third to the sixth decade, while in the seventh, horizontal orientation became more frequent than oblique. Changes in steep axis orientations through age decades could be by an actual switch from vertical orientations towards an oblique and, lastly, horizontal orientation without major changes in magnitude (axis rotation theory). They could also be explained by a magnitude decrease of a pre-existing vertical steep axis towards zero (spherical cornea) and then a progressive increase to a horizontal steep axis [35] (regression theory), or by a simple increase to a horizontal steep axis from a spherical cornea (low astigmatism progression). All these changes have been related to increasing eyelid pressure over the cornea [35] and imply a refractive shift from WTR to ATR for RA, as described by other authors [32,35].

Predicting PCA axis orientation in the overall astigmatic population would be quite accurate, as 93% of the subjects (between 68% and 96.1% based on studies from Supplemental Table S3) had a vertical steep posterior astigmatism axis. This could be improved when observing ACA axis orientation, as 99% of vertical ACA was also followed by a vertical PCA axis orientation. For the oblique ACA axis orientation, 85% still had vertical PCA axis orientation and for the horizontal ACA axis orientation, vertical PCA axis orientation dropped to 54% as another author also described [11]. Furthermore, for the right eyes in this study, the ACA axis orientations of the nasal region (0° to 90°) had more dispersion than the temporal regions (90° to 180°). For the horizontal ACA axis range of 0° to 30°, it seems impossible to predict PCA axis orientation due to its dispersion. Regarding the vertical ACA axis orientation, which has the best concordance with PCA axis orientation (also vertical), the ACA axis range of 90° to 120° had less dispersion (better predictability) than the ACA axis range of 60° to 90°.

Differences in other parameters between axis orientations were also studied. Patients with vertical ACA axis orientation were 10.53 years younger than those with horizontal, with no differences between the oblique and the other axis orientations. In addition, the vertical PCA axis orientation was 6.42 years younger than the oblique, with no differences between the horizontal and the other axis orientations. Another interesting finding was a 0.273 mm greater WTW in patients with vertical ACA axis orientation, with no differences between the oblique and the other two orientations. A wider WTW could better fit a flatter horizontal corneal curvature, resulting in a vertically steeper corneal curvature (or vertical axis orientation). Interestingly, these differences in WTW were not seen for PCA axis orientation, which could mean that there is no relation between the horizontal cornea width and PCA fitting as previously hypothesized, or that the WTW measurement is not the appropriate parameter to relate and it would correlate more with a deeper parameter as the angle-to-angle distance. Anterior corneal elevations were also greater in vertical ACA and PCA axis orientations (especially at the thinnest point), as some authors have previously described [36]. This is important to consider when using them on ectasia screening, [29] as normal anterior corneal elevations have been previously noted to have minimal variations (−0.1 to 3 µm) despite differences in age, refraction and race of the populations studied [37]. Lastly, patients with vertical ACA axis orientation had a lower RA but greater PCA magnitude than other axis orientations as other authors also found [13]. On the other hand, vertical PCA axis orientation had a greater RA than oblique orientation and greater PCA magnitude than other axis orientations. None of the ACA nor PCA axis orientations had differences in ACA magnitude, most probably due to the differences in subgroups sizes. On the contrary, Mendes detected a lower simulated keratometry (SimK) astigmatism magnitude for the oblique axis orientation compared to the vertical and horizontal axis orientations [13].

Some limitations in the present study should be acknowledged. Firstly, the effect of age can only be inferred as only longitudinal studies are specifically designed to assess this. Secondly, any case of subclinical ectasia could have been included in the study because there are not still definite topographic criteria to exclude ectasia, specifically at a younger age (they have more probability to have a non-detectable ectasia). Additionally, the examination was carried out from morning to afternoon, so daytime variations in corneal thickness have not been considered.

Regarding PCA evaluation, additional limitations have to be assumed when evaluating those measurements. First, data may be affected by the optical properties of the anterior cornea as the light passes through it first. The second limitation is that the main technologies that evaluate PCA use multiple b-scan images captured during a few seconds to re-compound the posterior cornea, so micro-movement artefacts are expected.

## 5. Conclusions

In summary, we hereby present an extensive normative database of ACA and PCA in Spanish individuals. In the population studied, ACA and PCA are, in most cases, oriented with the steep axis vertical. Steep axis orientations distribution was related to age and astigmatism magnitude and presented some differences with age, WTW, anterior corneal elevations and PCA magnitude.
Key findingsMean PCA was similar between different populations.ACA vertical steep axis orientation increased with less positive sphere, increasing ACA magnitude and younger age.PCA vertical steep axis only increased with increasing PCA magnitude.

## Figures and Tables

**Table 1:** Descriptive analysis of the variables evaluated.

Variables (Units)	Mean	SD	Min.	Max.
Age (y)	50.4	14.9	17.1	93.1
Sphere (D)	−0.44	3.47	−14.50	10.25
RA (D)	0.86	0.90	0	6
PD (mm)	3.01	0.66	1.65	8.49
ACD (mm)	2.80	0.43	1.52	4.09
Ele F Apex (µm)	1.80	1.16	−2	5
Ele F Thin (µm)	1.98	1.74	−7	10
Ele B Apex (µm)	3.48	3.35	−11	16
Ele B Thin (µm)	7.68	5.67	−6	28
Pachy (µm)	546.5	33.0	440	686
ACA (D)	1.01	0.79	0	5.80
PCA (D)	0.34	0.17	0	1.10
K1F (D)	43.34	1.51	37.60	48.20
K2F (D)	44.35	1.49	37.80	49.80
KmF (D)	43.84	1.45	37.70	49.00
K1B (D)	−6.19	0.25	−6.90	−5.30
K2B (D)	−6.53	0.28	−7.50	−5.40
KmB (D)	−6.35	0.25	−7.10	−5.30
QF	−0.31	0.12	−0.84	0.33
QB	−0.28	0.21	−0.97	0.60
WTW (mm)	12.09	0.42	10.90	13.40
AXL (mm)	23.92	1.56	20.24	32.59
Max = maximum, Min = minimum and SD = standard deviation				

**Table 2:** ACA and PCA measurements from the literature.

Author, Year	n (Eyes)	Measured Area	ACA (D)	PCA	Device	Race
Hoffman, 2010 [23]	23,239	2.5 mm	0.98 ± 0.78	n/a	IOL Master	Caucasic
Hwang, 2013 [24]	958	3 mm	1.35 ± 0.72	n/a	Pentacam	Korean
Atchison, 2008 [27]	106	3 mm	0.80 ± 0.41	0.33 ± 0.12	Pentacam	Caucasic
Tonn, 2015 [12]	3818	3 mm	1.15 ± 0.90	0.33 ± 0.18	Pentacam HR	Caucasic
Koch, 2012 [9]	715	4 mm	1.08 ± 0.71	0.30 ± 0.15	Galilei G2	n/a
Ueno, 2015 [25]	418	3 mm	1.05 ± 0.68	0.31 ± 0.14	Cassia SS-1000	Japanese
Ventura, 2022 [26]	3253	4 mm	1.35 ± 1.00	0.34 ± 0.15	Galilei G6	Brazilian
Nemeth, 2014 [10]	827	3 mm	0.9	0.3	Pentacam HR	Hungarian
Mendes, 2021 [13]	400	3 mm	1.21 ± 0.94	0.37 ± 0.24	Cassini	Portuguese
Present study	795	3 mm	1.01 ± 0.79	0.34 ± 0.17	Pentacam	Caucasic

## Data Availability

The data presented in this study are available on request from the corresponding author. The data are not publicly available due to privacy restrictions.

## Supplementary Materials

The following supporting information can be downloaded at: https://www.mdpi.com/article/10.3390/jcm12113664/s1, Figure S1: (A–C): Histogram of the RA (A), ACA (B) and PCA (C) with their Weibull density distribution curves. ACA = anterior corneal astigmatism; PCA = posterior corneal astigmatism; RA = Refractive astigmatism; Table S1: ANOVA test for ACA steep axis orientation; Table S2: ANOVA test for PCA steep axis orientation; Table S3: Steep axis orientation distribution for ACA and PCA from the literature.
