# Supplementary material for: Normative Topographic Anterior and Posterior Corneal Astigmatism: Axis Distribution and Its Relations with Ocular and Biometric Parameters
Source: J Clin Med. 2023 May 25;12(11):3664. doi: 10.3390/jcm12113664 (PMC10253972; doi:10.3390/jcm12113664)
Supplement: Supplementary file 1 [file jcm-12-03664-s001.zip › jcm-2327982-supplementary.pdf]

Supplemental Table S1.- ANOVA test for ACA steep axis orientation:

	ACA steep axis orientation						
	Vertical		Oblique		Horizontal		
Variables (units)	Mean	SD	Mean	SD	Mean	SD	Sig.
Age (y)	48,68$_a$	14,55	54,45$_b$	14,01	56,11$_b$	15,36	<0.000*
PD (mm)	3,04$_a$	0,68	2,95$_a$	0,63	2,94$_a$	0,58	0,210
ACD (mm)	2,81$_a$	0,43	2,74$_a$	0,45	2,76$_a$	0,42	0,179
WTW (mm)	12,11$_a$	0,41	12,07$_{a,b}$	0,43	11,97$_b$	0,40	0,009
AXL (mm)	23,91$_a$	1,52	23,92$_a$	1,68	23,95$_a$	1,68	0,981
Ele F Apex (μm)	1,91$_a$	1,13	1,49$_b$	1,20	1,51$_b$	1,20	<0.000*
Ele F Thin (μm)	2,19$_a$	1,74	1,53$_b$	1,56	1,23$_b$	1,65	<0.000*
Ele B Apex (μm)	3,32$_a$	3,35	4,00$_a$	3,11	3,82$_a$	3,61	0,084
Ele B Thin (μm)	7,47$_a$	5,64	7,87$_a$	5,20	8,73$_a$	6,30	0,116
Pachy (μm)	545,96$_a$	32,38	548,88$_a$	32,28	547,19$_a$	37,68	0,675
Sphere (D)	-,60$_a$	3,43	-,03$_a$	3,82	,02$_a$	3,18	0,106
RA (D)	,78$_a$	0,89	1,04$_b$	0,92	1,15$_b$	0,82	<0.000*
ACA (D)	1,11$_a$	0,82	,76$_b$	0,60	,71$_b$	0,60	<0.000*
PCA (D)	,37$_a$	0,17	,27$_b$	0,13	,23$_c$	0,13	<0.000*
K1F (D)	43,32$_a$	1,51	43,39$_a$	1,48	43,43$_a$	1,57	0,753
K2F (D)	44,43$_a$	1,49	44,15$_a$	1,41	44,14$_a$	1,56	0,058
KmF (D)	43,86$_a$	1,44	43,76$_a$	1,42	43,78$_a$	1,54	0,718
K1B (D)	-6,18$_a$	0,24	-6,21$_a$	0,26	-6,23$_a$	0,27	0,066
K2B (D)	-6,55$_a$	0,27	-6,48$_b$	0,27	-6,46$_b$	0,31	0,001*
KmB (D)	-6,36$_a$	0,24	-6,35$_a$	0,26	-6,34$_a$	0,29	0,804
QF	-,32$_a$	0,12	-,27$_b$	0,12	-,27$_b$	0,14	<0.000*
QB	-,27$_a$	0,22	-,31$_a$	0,20	-,30$_a$	0,22	0,117

Values in the same row and subtable that do not share the same subscript are significantly different at p< 0.05 in the two-sided test for equality for Column Means.
Cells without a subscript are not included in the test. The tests assume equal variances.[1]

[1.] Tests are adjusted for all pairwise comparisons within a row of each innermost subtable using the Bonferroni correction.

Supplemental Table S2.- ANOVA test for PCA steep axis orientation:

	PCA steep axis orientation						
	Vertical		Oblique		Horizontal		
Variables (units)	Mean	SD	Mean	SD	Mean	SD	Sig.
Age (y)	50,09$_{a}$	14,78	56,51$_{b}$	14,76	48,64$_{a,b}$	16,69	0,023*
PD (mm)	3,02$_{a}$	0,66	2,92$_{a}$	0,70	3,04$_{a}$	0,48	0,636
ACD (mm)	2,80$_{a,b}$	0,43	2,67$_{a}$	0,41	3,02$_{b}$	0,59	0,036*
WTW (mm)	12,09$_{a}$	0,41	11,99$_{a}$	0,39	12,01$_{a}$	0,68	0,268
AXL (mm)	23,92$_{a}$	1,55	23,63$_{a}$	1,69	24,90$_{a}$	1,29	0,070
Ele F Apex (µm)	1,82$_{a}$	1,17	1,36$_{b}$	1,08	1,91$_{a,b}$	0,70	0,039*
Ele F Thin (µm)	2,06$_{a}$	1,73	,79$_{b}$	1,49	1,27$_{a,b}$	1,68	0,000*
Ele B Apex (µm)	3,54$_{a}$	3,23	2,79$_{a}$	4,55	2,27$_{a}$	5,62	0,178
Ele B Thin (µm)	7,62$_{a}$	5,58	8,67$_{a}$	6,54	8,27$_{a}$	8,20	0,478
Pachy (µm)	546,07$_{a}$	32,63	553,24$_{a}$	39,84	551,73$_{a}$	31,73	0,342
Sphere (D)	-,47$_{a}$	3,46	,30$_{a}$	3,68	-1,55$_{a}$	2,32	0,227
RA (D)	,84$_{a}$	0,86	1,05$_{a,b}$	1,30	1,63$_{b}$	0,94	0,008*
ACA (D)	1,01$_{a}$	0,79	,89$_{a}$	0,63	1,43$_{a}$	1,05	0,128
PCA (D)	,35$_{a}$	0,17	,20$_{b}$	0,14	,20$_{b}$	0,13	0,000*
K1F (D)	43,34$_{a}$	1,52	43,50$_{a}$	1,32	42,85$_{a}$	1,13	0,445
K2F (D)	44,35$_{a}$	1,50	44,39$_{a}$	1,29	44,28$_{a}$	1,10	0,973
KmF (D)	43,84$_{a}$	1,46	43,94$_{a}$	1,27	43,55$_{a}$	0,99	0,723
K1B (D)	-6,19$_{a}$	0,25	-6,20$_{a}$	0,24	-6,06$_{a}$	0,36	0,241
K2B (D)	-6,54$_{a}$	0,28	-6,40$_{b}$	0,25	-6,27$_{b}$	0,36	0,000*
KmB (D)	-6,36$_{a}$	0,25	-6,30$_{a,b}$	0,24	-6,16$_{b}$	0,35	0,012*
QF	-,31$_{a}$	0,13	-,26$_{b}$	0,11	-,31$_{a,b}$	0,10	0,045*
QB	-,28$_{a}$	0,21	-,21$_{a}$	0,30	-,24$_{a}$	0,26	0,104

Values in the same row and subtable that do not share the same subscript are significantly different at p< 0.05 in the two-sided test for equality for Column Means.
Cells without a subscript are not included in the test. The tests assume equal variances.[1]

[1.] Tests are adjusted for all pairwise comparisons within a row of each innermost subtable using the Bonferroni correction.

Supplemental Table S3.- Steep axis orientation distribution for ACA and PCA from the literature.

Authors, year	Steep ACA axis			Steep PCA axis		
	**Vertical** WTR	**Oblique** Obl	**Horizontal** ATR	**Vertical** ATR	**Oblique** Obl	**Horizontal** WTR
**Ho, 2009 [7]**	71,8	13,2	15	96,1	1,9	2
**Nemeth, 2014 [10]**	65,8	16,4	17,8	88,7	8,2	3,1
**Mendes, 2021 [13]**	47	16	37	68	19	13
**Present study**	73,5	14,2	12,3	93,3	5,3	1,4
**Savini, 2014 [11]**	84	4,5	11,5	93	n/a	n/a
**Koch, 2012 [9]**	50,9	n/a	n/a	86,8	n/a	n/a
**Ventura, 2022 [26]**	74,5	n/a	n/a	93,1	n/a	n/a
**Tonn, 2015 [12]**	71,2	n/a	n/a	88,8	n/a	n/a
**Ueno, 2015 [25]**	63,6	10,6	25,8	n/a	n/a	n/a
**Hoffman, 2010 [23]**	46,8	18,9	34,3	n/a	n/a	n/a
**Schuster, 2017 [32]**	70,8	14,5	14,7	n/a	n/a	n/a

n/a: not available

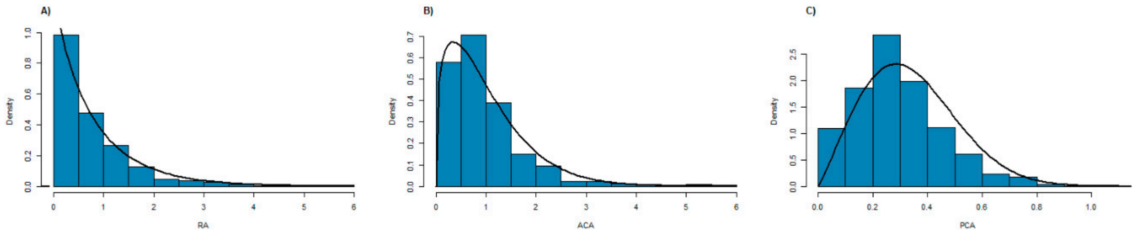

Figure S1. (A–C): Histogram of the RA (A), ACA (B) and PCA (C) with their Weibull density distribution curves. ACA = anterior corneal astigmatism; PCA = posterior corneal astigmatism; RA = Refractive astigmatism.
